# Intervention not always necessary in post-appendectomy abscesses in children; clinical experience in a tertiary surgical centre and an overview of the literature

**DOI:** 10.1007/s00431-016-2756-0

**Published:** 2016-08-10

**Authors:** Ramon R. Gorter, Suzanne Meiring, Johanna H. van der Lee, Hugo A. Heij

**Affiliations:** 1 Paediatric Surgical Centre of Amsterdam, Emma Children’s Hospital AMC and VU University Medical Centre, P. O. Box 22660, 1100 DD Amsterdam, The Netherlands; 2 Department of Surgery, Red Cross Hospital, Beverwijk, the Netherlands; 3 Division Woman and Child, Academic Medical Centre, Amsterdam, the Netherlands

**Keywords:** Appendicitis, Drainage procedure, Intra-abdominal abscess, Post-appendectomy abscess

## Abstract

This study aims to provide an overview of both our own experience and the available literature on the treatment of post-appendectomy abscess (PAA) in children. We performed a historical cohort study encompassing all children aged 0–17 years old treated for a radiologically confirmed PAA between 2007 and 2013. Their medical charts were reviewed and descriptive analyses were performed. A literature search on the treatment of PAA in children was performed. In our cohort, 25 out of 372 (7 %) children developed a PAA. Thirteen were treated with a noninvasive strategy and 12 with an invasive strategy (percutaneous or surgical drainage). The immediate success rate was 9/13 (69 %) and 8/12 (67 %) for the noninvasive and invasive strategy, respectively. In both groups, four children (31 and 33 % resp.) required delayed interventions after their initial treatment. In the literature review, six studies were included which reported a median (range) frequency of persistent or recurrent abscess of 9 % (0–30 %), 50 % (0–100 %) and 24 % (0–33 %) for the antibiotic (noninvasive), percutaneous drainage (invasive) and surgical drainage strategies, respectively.

*Conclusion*: Although confounding by indication cannot be excluded, we recommend noninvasive treatment as a safe strategy for PAA in children with stable condition.

**Table:** 

**What is known:** • *Post-appendectomy abscess is a well-known and feared complication, occurring in up to 24 % of the children treated surgically for appendicitis*. • *Several strategies are available to treat this condition, all with advantages and disadvantages*.
**What is new:** • *Noninvasive strategy is a safe strategy for children with a PAA in a stable condition.* *• An overview of the literature (the first to our knowledge) supports the above-mentioned statement*.

## Introduction

Each year, approximately 5500 appendectomies (34 % of the total amount of appendectomies) for acute appendicitis are performed in the Netherlands for patients younger than 20 years old [2]. Although highly effective, infectious complications such as superficial site infection and post-appendectomy abscesses (PAA) can occur. These complications are associated with readmission and may require additional intervention [15]. Although their incidence has been reduced since the systematic use of prophylactic antibiotics, it still ranges from 1 to 24 % depending on the severity of the appendicitis and the surgical approach [11–13, 18]. An old surgical dogma mandates that pus should always be drained from the body. Historically, PAA were therefore treated by drainage, either surgically (open or laparoscopic) or percutaneously under radiological guidance. It has been observed that noninvasive treatment (with antibiotics or even without antibiotics but with close clinical monitoring) is also effective in many cases [1, 4–6]. Choice of treatment not only depends on several factors including clinical, biochemical and radiological characteristics but also on preferences of individual surgeons. The aim of this paper is to provide an overview both of our own experience and the available literature on the treatment of post-appendectomy abscesses in children.

## Materials and methods

This study encompasses two elements: a retrospective cohort study as well as a systematic review of the current literature. The methods of both elements will be discussed in consecutive order in this section.

### Retrospective cohort study

Retrospectively, we have selected patients from our database encompassing all children aged 0–17 years old treated for acute appendicitis in our tertiary referral centre. Patients were eligible if they had been treated for a radiologically confirmed post-appendectomy abscess between January 2007 and December 2013. The definition for a PAA was as follows: “accumulation of purulent fluid in a walled-off space within the abdominal cavity after an appendectomy seen on ultrasound, CT scan or MRI with concomitant clinical and biochemical signs of infection”. Patients with clinical suspicion for a post-appendectomy abscess but without radiological confirmation and patients in whom the initial appendectomy had not been performed in our centre were excluded as we wanted to investigate our own cohort, and in most cases of transferred patients essential data was missing.

#### Initial appendectomy

Both the laparoscopic and open approach for an appendectomy is used in our centre. In all cases, antibiotic prophylaxis is administered 30 min prior to incision. Intraoperatively and based on the pathological findings, the diagnosis of either simple (uncomplicated) or complex (complicated) appendicitis is made based upon the following predefined criteria:

- Simple

- A perioperative diagnosis made by the surgeon based on signs of an inflamed appendix without signs of gangrene, perforation, purulent fluid, contained phlegmon or IAA. There is no need for additional postoperative antibiotics (exception: indication for perioperative spillage) and
- Histopathology: confirmation of the diagnosis of appendicitis (infiltration of the muscularis propria by neutrophils without signs of necrosis or perforation)

- Complex

- A perioperative diagnosis made by the surgeon based on signs of a gangrenous appendix with or without perforation, intra-abdominal abscess, periappendicular contained phlegmon or purulent free fluid and the need for additional postoperative antibiotics directly after appendectomy or
- Histopathology: findings of extensive necrotic tissue in the outer layer of the appendix or signs of perforation

In case of complex appendicitis, broad-spectrum antibiotics (either the combination of amoxicillin/clavulanic acid (100/10 mg/kg/day) together with gentamicin (7 mg/kg/day) or cefuroxime (100 mg/kg/day) together with metronidazole (30 mg/kg/day)) are administered intravenously for 5 days. In case of simple appendicitis, no additional antibiotics are given postoperatively.

We used a standardized data extraction form to review the medical charts containing the following variables:

- Baseline: age (years), gender, type of appendicitis (complexity), temperature (degrees Celsius) at time of diagnosis of PAA, level of C-reactive protein (CRP) (mg/L) at time of diagnosis of PAA, surgical approach of appendectomy, abscess size (cm), abscess location and single/multiple abscesses
- Treatment: treatment strategy for PAA is divided in the following:

- Noninvasive strategy (with or without administration of antibiotics)
- Invasive strategy

- Percutaneous drainage strategy (with additional antibiotics)
- Surgical (laparoscopic or open) drainage strategy (with additional antibiotics)

- Complications after treatment of PAA: recurrent or persistent abscess (a radiologically confirmed abscess on the same or new location requiring additional intervention with clinical and biochemical signs of infection), iatrogenic perforation and secondary small bowel obstruction requiring an additional intervention or readmission.
- Additional interventions: need for additional antibiotics and/or drainage and/or ICU admittance, need for readmission
- Additional checkup: number of additional checkups or imaging studies (ultrasound, computed tomography (CT) scan, magnetic resonance imaging (MRI))
- Length of hospital stay

#### Statistical analysis

Descriptive analyses were performed using SPSS version 20 (IBM, Armonk, NY, USA).

### Literature review

In addition to our retrospective cohort study, we have performed a systematic literature review of the available evidence regarding the treatment of post-appendectomy abscesses.

#### Systematic literature search

We searched the PubMed database using the following search strategies: (“Appendectomy”[Mesh] or Appendectom*) and (“Abdominal Abscess”[Mesh] or Intra-Abdominal Abscess*[tiab] or Intra-abdominal Abscess*[tiab] or Abdominal Abscess*[tiab]) and (child*[tw] or schoolchild*[tw] or infan*[tw] or adolescen*[tw] or pediatri*[tw] or paediatr*[tw] or boy[tw] or boys[tw] or boyhood[tw] or girl[tw] or girls[tw] or girlhood[tw] or youth[tw] or youths[tw] or baby[tw] or babies[tw] or toddler*[tw]). For the primary search, there were no restrictions regarding language or date. The final search was conducted on the 5th of November 2014.

#### Study selection process

Two authors independently identified potential studies of interest based upon predefined inclusion and exclusion criteria. Studies were eligible if they focused on the treatment of post-appendectomy abscesses in children (aged 0–17 years old). We excluded studies focusing on the treatment of a periappendicular abscess and case reports. In addition, language was restricted to Dutch and English. Any disagreements were resolved by discussion or when necessary by consultation with a third reviewer.

#### Data extraction

Two authors independently extracted the data of the included papers. The following data were gathered:

- Study characteristics: year of publication, first author, type of study and number of patients with PAA included
- Treatment: treatment strategy for PAA
- Complications
- Additional interventions: need for additional antibiotics or drainage procedure

Again only descriptive analyses were performed using SPSS version 20 (IBM, Armonk, NY, USA).

The medical ethics committee of the VU University medical centre confirmed that no approval for this study was necessary by national law.

## Results

### General characteristics

During the study period, 372 children underwent an appendectomy in our centre of which 25 (7 % (95 % CI 5–10 %)) developed a post-appendectomy abscess. Diagnosis was made by ultrasound in all patients after postoperative antibiotic treatment was completed. None underwent a CT or MRI for the initial diagnosis of PAA. Twenty-three patients (92 %) were diagnosed with complex appendicitis. In 13 patients (52 %), the PAA was initially treated with a noninvasive strategy and in 12 patients invasive strategy was followed. The general characteristics of both groups are displayed in Table 1. Ten patients (77 %) in the noninvasive group did not receive any antibiotics for the PAA treatment. These patients were only monitored for any signs of clinical deterioration. The reason why a particular treatment was chosen was in the noninvasive group: approximately half of the patients could not be drained percutaneously (23 %) or had such a small size abscess (23 %) that a noninvasive strategy was chosen. In the other half (54 %), the clinical condition of the patient was so good that a noninvasive strategy was chosen.

**Table 1:** Baseline characteristics of the treatment groups

	Noninvasive *N* = 13	Invasive *N* = 12
Male gender	4 (31)	5 (42)
Age (years)^a^	9 (4–14)	10 (3–14)
Temperature (degrees Celsius)^a^	38.8 (38.4–39.5)	39.1 (38.5–40.0)
Type appendicitis (simple)	2 (15)	0
Time after appendectomy (days)^a^	8 (4–29)	8.5 (5–17)
CRP (mg/L)^a^	160 (35–345)	145 (62–400)
Initial surgical approach		
Open appendectomy	6 (46)	7 (58)
Laparoscopic appendectomy	7 (54)	5 (42)
Abscess size		
Unknown	1 (8)	0
Small (<3 cm)	2 (15)	0
Medium (3–6 cm)	2 (15)	3 (25)
Large (>6 cm)	4 (31)	3 (25)
Multiple	4 (31)	6 (50)
Abscess location		
Right lower quadrant	7 (54)	4 (33)
Douglas space	2 (15)	1 (8)
Right upper quadrant	0	1 (8)
Multiple	4 (31)	6 (50)

Results are displayed as number of patients (%) unless stated otherwise

^a^Results are displayed as median (minimum–maximum)

In the invasive group, the clinical condition mandated an intervention of invasive nature in all patients.

### Primary outcome

**Fig. 1:**
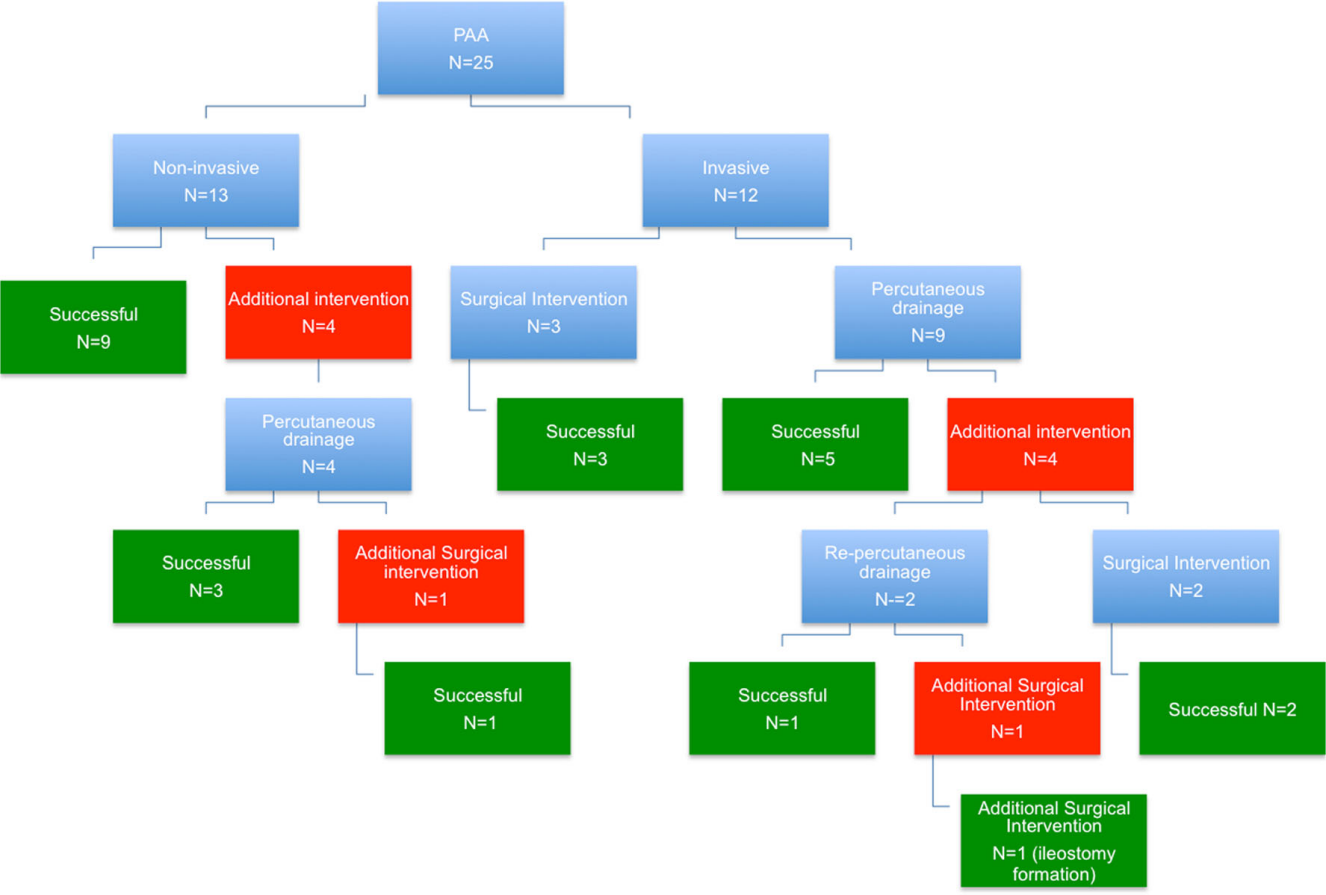
Flow diagram of the PAA treatment groups

Figure 1 displays the flow diagram of both treatment groups. Noninvasive treatment was successful in 9 out of 13 patients (69 %). Four patients underwent a delayed (secondary) intervention due to a persistent or recurrent abscess. Timing of the delayed intervention ranged from 1 to 13 days after diagnosis of PAA. All four patients underwent a percutaneous drainage under radiological guidance. One of them underwent a laparotomy 12 days after the percutaneous drainage due to the suspicion of stump leakage, which could not be confirmed during surgery. However, a persistent abscess was noted during surgery, which was evacuated. Postoperative course was uneventful.

In the invasive treatment group, 8 out of 12 (67 %) patients were successfully treated with their initial treatment strategy all without any complications. Four patients, all treated with percutaneous drainage, required an additional intervention. Two underwent re-percutaneous drainage due to persistent or recurrent abscesses 4 and 10 days after initial percutaneous drainage, respectively. One of them underwent a subsequent laparotomy due to an iatrogenic perforation 3 days after this additional procedure. Seven days later, he underwent a second laparotomy with the formation of an ileostomy due to persistent leakage.

The two others underwent a laparotomy after their initial drainage procedure. One patient underwent the laparotomy 14 days after his initial percutaneous drainage procedure for the suspicion of persistent abscesses and secondary bowel obstruction. His postoperative course was complicated by a splenic haemorrhage, superficial site infection and prolonged paralytic bowel obstruction for which parenteral feeding was needed. The other patient underwent a laparotomy 3 months after his drainage procedure for small bowel obstruction due to adhesions around the old drain track. His postoperative course was uneventful.

The total number of interventions was 5 (1 surgical and 4 percutaneous) in the noninvasive group versus 20 (6 surgical and 14 percutaneous) in the invasive group.

### Secondary outcomes

The results concerning the length of hospitalization, number of checkups and imaging per patient are displayed in Table 2. As expected, the shorter hospital stay (median 7 days) was in the noninvasive treatment group versus median 17 days in the invasive group. Ultrasound was the method of preference for follow-up.

**Table 2:** Hospitalization, checkups and imaging in both treatment groups

	Noninvasive *N* = 13	Invasive *N* = 12
Hospital stay (days)^a^	7 (1–22)	17 (1–42)
Number of outpatient checkups per patient^a^	1 (0–3)	1 (0–2)
Number of ultrasounds per patient ^a^	3 (1–8)	4 (0–8)
Number of CTs per patient^a^	0 (0–0)	0 (0–3)
Number of MRIs per patient^a^	0 (0–0)	0 (0–1)

^a^Results are displayed as median (minimum–maximum)

### Results of the literature search

**Fig. 2:**
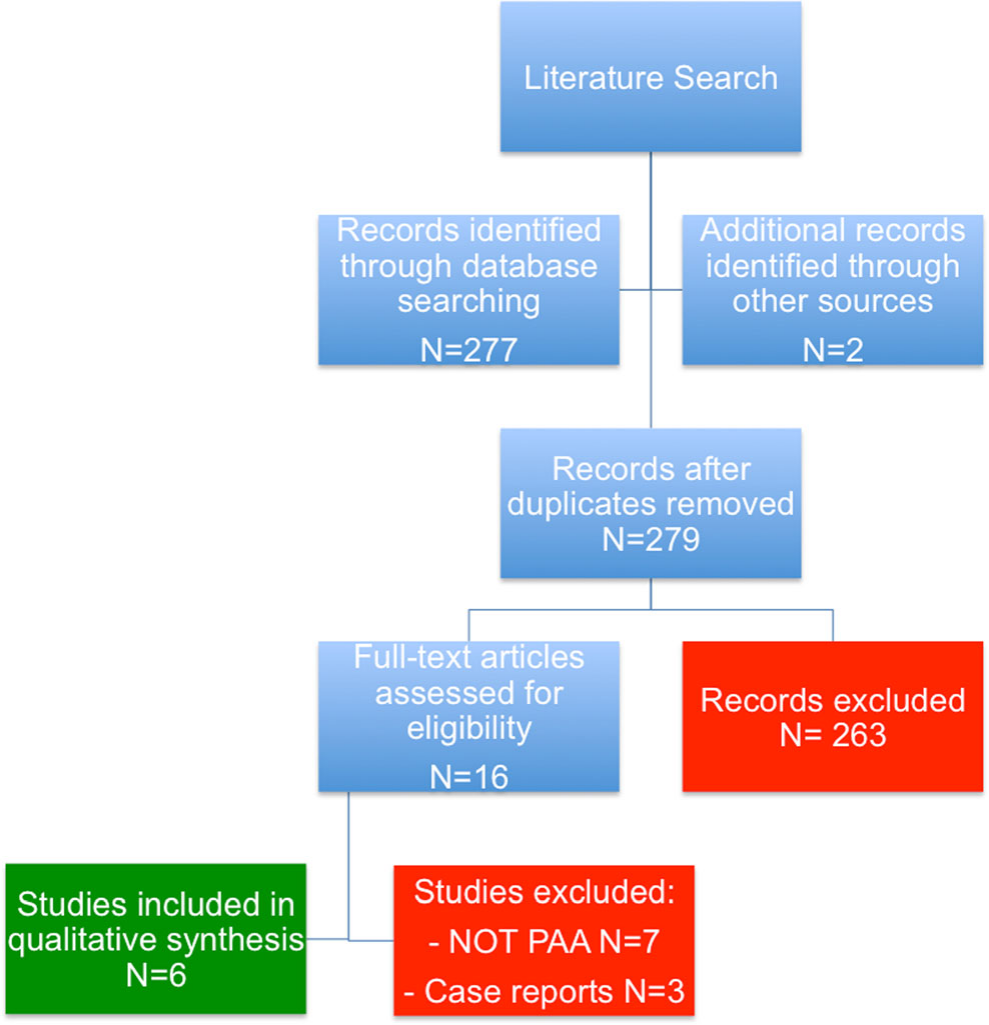
Flow diagram of the literature search

Figure 2 shows a flow diagram of the search results and selection of papers. A total of 279 studies were identified through the search, of which 263 articles were excluded after screening on title and abstract. Of the 16 remaining potentially relevant articles, full text was retrieved and eventually ten were excluded for the following reasons: seven articles did not address the post-appendectomy abscess and three articles were case reports. As a result, six articles were included in the review [3–7, 14].

### Description of the studies

Table 3 provides an overview of the six included studies [3–7, 14]. All were historical cohort studies (Oxford level 2b) containing small patient groups (ranging from 10 to 25) [3–8, 14].

**Table 3:** Description of included studies in this overview [3–7, 14]

Name–year	Type of study	Intervention: *N*	Persistent/recurrent abscess^a^	Additional intervention	Complication (number of patients)
Clark 2011 [3]	Historical cohort	Surgical: 12	3 (25)	Surgical: 3	Recurrent abscess: 2
					Fistula: 1
					Scrotal abscess: 1
Dhaou 2010 [4]	Historical cohort	Antibiotics: 7	1 (15)	Surgical: 1	Recurrent abscess: 1
		Radiological: 1	1 (100)	Surgical: 1	Persistent abscess: 1
		Surgical: 6	0		
Forgues 2007 [6]	Historical cohort	Antibiotics: 22	0		
		Radiological: 1	0		
		Surgical: 3	1 (33)	Surgical: 1	Persistent abscess: 1
Dobremez 2003 [5]	Historical cohort	Antibiotics: 11	1 (9)	Surgical: 1	Recurrent abscess: 1
					Gallbladder stone: 1
		Surgical: 11	2 (22)	Antibiotics: 6	Fistula: 4
				Surgical: 1	Recurrent abscess: 2
					Gas gangrene: 1
					Intra-peritoneal bleeding: 1
Okoye 1998 [14]	Historical cohort	Antibiotics: 23	2 (9)	Surgical: 1	Persistent abscess: 2
				Percutaneous: 1	Pleural effusion: 1
Gorenstein 1994 [7]	Historical cohort	Antibiotics: 10	3 (30)	Percutaneous: 2	Persistent abscess: 3
				Antibiotics: 1	

^a^Data is displayed as: Number of patients (percentage)

Three papers report about one intervention [3, 7, 14], one about two interventions [5] and two about three interventions [4, 6]. Treatment strategies of antibiotics, surgical drainage and radiological drainage were described in five, four and two studies, respectively. The median (range) reported frequencies of persistent or recurrent abscesses were 9 % (0–30 %), 50 % (0–100 %) and 24 % (0–33 %) for the antibiotics (noninvasive), radiological drainage (invasive) and surgical drainage groups (invasive), respectively [3–7, 14]. The most frequently reported additional intervention for a recurrent or persistent abscess was surgical drainage.

All patients who required an additional intervention after their initial noninvasive or invasive treatment only required one intervention. Of special interest is the study by Dobremez in which 11 children underwent surgical drainage for their PAA [5]. A relatively large number of complications (*n* = 7, 64 %) was noted in this group. Complications consisted of three patients with digestive fistulae, which closed spontaneously under triple antibiotic therapy and parenteral nutrition, two patients with recurrent intra-abdominal abscesses, which were treated non-operatively, one patient with parietal gas gangrene requiring surgery, antibiotics and hyperbaric oxygen therapy, and one patient with intra-peritoneal bleeding with a fistula of the small intestine requiring an additional surgical intervention [5]. In the 11 patients reported by Dobremez who had been treated with antibiotics, only two complications occurred; recurrent abscess in one and a gallbladder stone secondary to the antibiotics given in one, which did not require additional therapy [5].

## Discussion

Our study provides an overview of the noninvasive and invasive treatment strategies for post-appendectomy abscesses and their results in our own centre and in the literature. In our centre, noninvasive treatment was chosen mainly due to the good clinical condition of the patient and was successful in 69 % of the patients. In total, 5 invasive procedures were needed in 4 patients of this group of 13. A total of 20 invasive procedures were performed in 12 patients for whom noninvasive treatment was considered contraindicated.

The treatment of PAA remains subject of debate and unequivocal guidelines are lacking. The Society for Surgery in the Netherlands advocates a noninvasive strategy (with or without antibiotics) for PAA in children, whereas in adults, they recommend percutaneous drainage [1]. In a comprehensive search of the literature, we only found historical cohort studies describing the results of various treatment options for PAA in children [3–7, 14]. RCTs and systematic reviews are lacking. Some surgeons are still reluctant to treat post-appendectomy abscesses with a noninvasive strategy. Therefore, an RCT on this topic would probably not be feasible.

Drainage of intra-abdominal abscesses has often been recommended in the past. Both surgical and radiologically guided percutaneous drainage procedures have been described in the literature. These are invasive procedures that require some form of anaesthesia in children and they are associated with significant morbidity making them a less attractive option [3, 5]. Iatrogenic perforations of the bowel, fistula formation and major haemorrhage have all been reported after radiologically guided percutaneous drainage [5, 9]. Moreover, radiologically guided percutaneous drainage is not suitable for all patients with PAA. The number of abscesses and their location determines whether or not radiological drainage is an option. Noninvasive treatment strategy was chosen in six patients in our cohort due to the relatively small size of the abscess or due to the fact that percutaneous drainage would not be feasible. Surgical drainage of post-appendectomy abscesses is successful in most cases but it also causes significant morbidity (up to 64 %) as demonstrated by Dobremez [5]. In our own cohort, the three patients who underwent surgical drainage of their PAA were all treated successfully without complications. We hypothesize that this might be due to the fact that in all our patients a laparotomy with excellent visualization of the entire abdomen with meticulous suction leaving no pus behind was achieved. Alternatively, laparoscopic drainage can be undertaken. Clark et al. reported promising results of this technique and it deserves further investigation [3].

Favourable results of noninvasive treatments with or without antibiotics have been reported in the literature [4–7, 14]. In our centre, we saw a low recurrent or persistent abscess rate and only 4 of the 13 patients required a total of 5 delayed interventions, which is in line with current literature [4–7, 14]. In our opinion, this strategy is successful and is to be preferred to the invasive treatment strategy. However, contraindications for this strategy such as signs of sepsis or even septic shock should be kept in mind. Furthermore, frequent reassessment of the clinical, biochemical and even radiological status is recommended.

Some authors also mention that the choice of treatment strategy for PAA depended on the size of the abscess. They state that only small abscesses (<3 cm) should be treated with noninvasive options whereas larger ones should be drained by the radiologist [10, 16, 17]. In our cohort, we have also treated patients with larger (more than 6 cm) and multiple abscesses with noninvasive strategy successfully. Forgues et al. have also treated larger abscesses with noninvasive treatment [6]. In our opinion, the large size of the abscesses might therefore not be a contraindication for noninvasive treatment, although this is based on a relatively low number of patients.

Our study has several limitations. Due to the retrospective nature, it is prone to information bias as it relies on the accuracy of the medical charts. Secondly, the surgeon on call made the decision for the treatment strategy of the PAA.

Furthermore, the choice whether to drain the PAA radiologically or surgically depends on factors such as experience of the radiologist, location and number of abscesses. Therefore, confounding by indication is inevitable.

Although the two groups are not comparable, it is clear that in a selected group of patients with PAA, noninvasive treatment, even without antibiotics, can result in resolution of the abscess and cure of the patient without the need for interventions.

Prospective studies should focus on the indications for either noninvasive treatment, with or without antibiotics, or invasive treatment.

In summary, based on this overview of our experience and the literature of the treatment of PAA in children, we conclude that noninvasive treatment is a safe strategy for PAA in children with stable condition.

## Abbreviations

CRP: C-reactive protein
CT-scan: Computed tomography
MRI: Magnetic resonance imaging
PAA: Post-appendectomy abscess
